# Cronkhite-Canada Syndrome: Gastric Involvement Diagnosed by MDCT

**DOI:** 10.1155/2009/148795

**Published:** 2009-08-04

**Authors:** Jonathan D. Samet, Karen M. Horton, Elliot K. Fishman, Christine A. Iacobuzio-Donahue

**Affiliations:** ^1^Department of Radiology, Feinberg School of Medicine, Northwestern University, 676 North Saint Clair Street, Suite 800 Chicago, IL 60611, USA; ^2^Department of Radiology, Johns Hopkins Hospital, Johns Hopkins Medical Institutions, JHOC 3253, 601 North Caroline Street, Baltimore, MD 21287-0801, USA; ^3^Department of Pathology, Johns Hopkins Hospital, Johns Hopkins Medical Institutions, CRB II 343, 1550 Orleans Street, Baltimore, MD 21231, USA

## Abstract

Chronkhite-Canada is a rare nonfamilial polyposis syndrome that usually presents as chronic malabsorption in adults. We present a case of a-73-year old woman with chronic gastrointestinal bleeding and malnutrition. On CT imaging she was found to have massive gastric polyps, which on biopsy was most consistent with Cronkhite-Canada syndrome.

## 1. Introduction

Since its initial description in 1955, there have been almost 400 reported cases of Cronkhite-Canada syndrome (CCS) [1]. It is a sporadic polyposis syndrome of the gastrointestinal tract which has features resembling both Juvenile Polyposis syndrome (JPS) and Peutz-Jeghers syndrome (PJS) [2, 3]. The polyps can interfere with absorption producing chronic malnutrition. Patients typically exhibit characteristic clinical findings of diarrhea, hypogeusia, nail dystrophy, and alopecia [4]. We present an interesting case of a-73-year old woman with chronic malnutrition and gastrointestinal bleeding. An abdominal CT was performed which demonstrated a markedly distended stomach due to mass-like fold thickening and areas of hemorrhage. Subsequent biopsy revealed inflamed and edematous gastric epithelium with associated prominent foveolar hyperplasia. This was most consistent with CCS.

## 2. Case Report

The patient is a-73-year old female with an extensive past medical history. The patient first reports a diagnosis of colonic polyposis at age 48 after presenting with hematochezia. Over the subsequent 25 years, the patient had undergone repeated colonoscopy with removal of numerous polyps. By her report, some of the polyps were adenomatous, but the outside records were no longer accessible. For several years, the patient carried the diagnosis of familial adenomatous polyposis. The patient began experiencing increased weight loss and bleeding and therefore underwent subtotal colectomy with ileorectal anastomosis five years prior to this admission at an outside hospital. Pathology at the outside facility revealed approximately 50 juvenile polyps most consistent with Juvenile Polyposis syndrome. The patient's mother and half brother reportedly died of colon cancer, both in their 50s.

**Figure 1:**
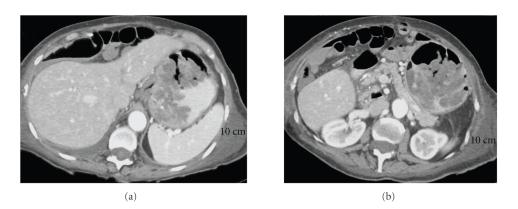
(a) Axial CT through the gastric cardia demonstrates large lobular folds forming a mass-like configuration. (b) Axial CT through the gastric body shows a distended stomach with multiple lobular mass-like folds.

**Figure 2:**
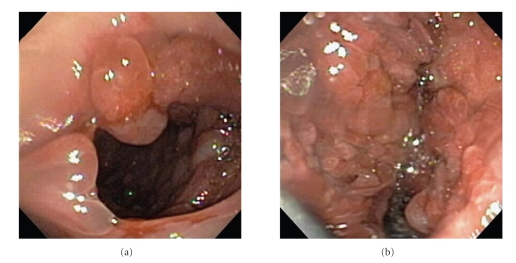
(a) Image obtained during upper endoscopy of the stomach demonstrates multiple polyps and edematous mucosa. (b) Image obtained during upper endoscopy of the stomach demonstrates numerous polyps which coalesce, giving a mass-like appearance.

**Figure 3:**
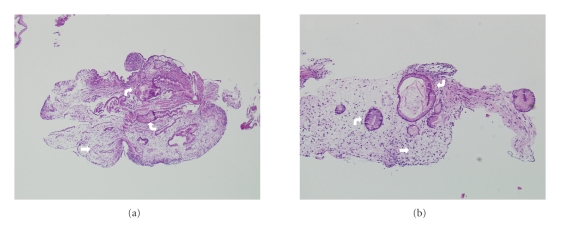
Histopathology of gastric mucosa. (a) Gastric polyp with hyperplastic features of foveolar epithelium (curved arrows) in a background of prominent lamina propria edema and inflammation (straight arrow). (b) Nonpolypoid gastric mucosa. Similar features are present; hyperplastic epithelium (curved arrows) and lamina propria edema (straight arrow) as in Panel (a).

The patient presented to our hospital with recurrent melanotic diarrhea and anemia. Initial physical exam in the emergency room did not mention any alopecia, cutaneous hyperpigmentation, or dystrophic nails. A CT of the abdomen with IV and oral contrast revealed a markedly distended stomach due to mass-like fold thickening and areas of hemorrhage (Figure 1). A small bowel series was also performed which showed that the stomach was markedly dilated with multiple filling defects and ulceration causing an asymmetrical narrowing of the lumen. There was no evidence of gastric outlet obstruction. Upper endoscopy showed large mucinous fungating friable folds and numerous polyps of various sizes in the fundus and proximal body of the stomach (Figure 2). The masses were difficult to differentiate into separate lesions. The appearance of the mucosa of both the polyps and the polypoid masses appeared highly cystic and erythematous. There was no active bleeding during endoscopy. Sigmoidoscopy found and biopsied three rectal polyps. Biopsies of the lesions from the stomach and rectum both showed inflamed and edematous epithelium with associated prominent foveolar hyperplasia (Figure 3). Upon review of the outside slides from her colectomy, taken together with the current biopsies, it was determined in retrospect that these findings were actually most consistent with polyps that arise in the setting of CCS, rather than juvenile polyposis.

During her hospitalization at our institution, her diarrhea showed marked cessation after the administration of antibiotic (amoxicillin) and corticosteroid therapy and pantoprazole. Patient also received multivitamins. For her malnutrition, she was given TPN, and her symptoms improved. Surgery, although initially considered at presentation, was deferred given the patients continued improvement and the revised diagnosis of CCS. Patient was scheduled for follow-up at out polyposis clinic but did not keep her appointments and was lost to follow-up.

## 3. Discussion

Cronkhite-Canada syndrome is a nonfamilial polyposis syndrome characterized by hamartomatous polyps in the stomach, small bowel, and colon. Esophageal involvement is very rare [5, 6]. Hamartoma refers to aberrant growth of non-neoplastic tissue that is normally present in a particular region. The polyps of CCS are small and are more commonly sessile than pedunculated. [5, 6] Typically the stomach has thickened rugal folds, and the polyps which are small to moderate in size are said to carpet the mucosa. The small bowel contains multiple small polyps. In the rectum and colon, the polyps are diffuse, but the mucosa is not involved as much as in the stomach. [5, 6] Microscopically, the involved tissue is edematous and inflamed.

CCS is rare. Less than 400 cases have been reported in the world literature [7]. It usually occurs in older people around the sixth decade of life [2]. Most of the reported cases are found in Japan, for an unknown reason [2]. An epidemiological study of the clinical features found that 35.4% of people presented with diarrhea, 40.9% with hypogeusia, 8.2% with alopecia, 6.4% with xerostomia, and 9.1% with abdominal discomfort [8]. 

Patients with CCS are very ill, suffering from the sequelae of malabsorption. They are severely malnourished, leading to anemia, electrolyte disturbances, and hypoproteinemia. The patients usually die from gastrointestinal bleeding, sepsis, and heart failure. The 5-year survival rate is grim at 55% [9]. The pathogenesis is still unknown at this time, although some authors have suggested an autoimmune process [10]. 

Although no medical therapy is curative, some therapeutic modalities have demonstrated improvement in symptoms and a decrease in polyp burden. For example, reports have shown that by correcting the nutritional deficits via repleting minerals, vitamins, enteral supplements, and parenteral nutrition if necessary, some patients showed improvement in alopecia, hyperpigmentation, onychodsytrophy, and polyp burden. Oral corticosteroids have also been shown to reduce symptoms related to CCS. H2 blockers such as ranitidine can decrease the number of polyps seen on endoscopy. Ward and Wolfsen hypothesized that mast cells may play a role in the development of CCS and accordingly described a patient who improved with cromolyn sulfate, a mast cell stabilizer. Specifically, they used a combination therapy including cromolyn sulfate, H1 and H2 blockers, corticosteroids, and antibiotics, which induced remission in their patient [11, 12]. Okamoto et al. presented a patient with CCS in whom the nutritional status, ectodermal findings, and polyp burden improved using an anti-Helicobacter pylori regimen of clarithromycin, amoxicillin, and lansoprazole [13]. 

The role of surgery in the treatment of CCS is undefined. Some reports have described patients with symptomatic remissions after resection of a diffusely involved segment of bowel. However, some authors argue that surgery for CCS should only be utilized in cases of severe gastrointestinal complications [12].

The differential diagnosis includes other polyposis syndromes such as Peutz-Jeghers syndrome, Juvenile Polyposis syndrome, and Turcot syndrome. It has been suggested that PJS, JPS, and CCS are all different variations of the same disease [2]. PJS and JPS have a proven genetic component, whereas CCS is still thought to be nonhereditary. CCS is pathologically distinguished from Juvenile Polyposis syndrome (JPS) in that the intervening mucosa between hamartomatous polyps is as edematous and inflamed as the polyp, whereas the intervening mucosa between juvenile polyps in JPS is normal. JPS generally presents early in life and CCS later in life, but there is overlap [2]. 

The malignant potential of polyps in CCS is unclear. Of the 387 cases reported by the end of 2002, 50 (13%) were associated with cancer. 31 (8%) were cases of colon cancer and, 19 (5%) were gastric cancer. The histology of these cancers is adenocarcinoma. It is still controversial whether these cancers are coincidental or if they begin as benign polyps and then develop malignant transformation. Yashiro et al. suggested the possibility of a precursor lesion to adenocarcinoma called a serrated adenoma that is much more common in CCS than in general polyps. In the cases of CCS they studied, both the serrated ademomas and the adenocarcinoma demonstrated microsatellite instability and overexpression of the p53 protein [7]. 

In conclusion, we presented a patient with features typical of Cronkhite-Canada syndrome. The CT findings are representative of the extensive gastric involvement seen in patients with this syndrome. As more cases are reported to literature, hopefully a better understanding of this fascinating entity will come to fruition. It is clear, as evidenced by this case report, gastrointestinal polyposis syndromes can sometimes be difficult to classify. This patient initially carried the diagnosis of familial adenomatous polyposis, then juvenile polyposis, and finally after extensive pathological review, CCS.
